# Moving the Target on the Optimal Adjuvant Strategy for Resected Pancreatic Cancers: A Systematic Review with Meta-Analysis

**DOI:** 10.3390/cancers12030534

**Published:** 2020-02-26

**Authors:** Antonio Galvano, Marta Castiglia, Sergio Rizzo, Nicola Silvestris, Oronzo Brunetti, Giovanni Vaccaro, Valerio Gristina, Nadia Barraco, Marco Bono, Giovanni Guercio, Giuseppa Graceffa, Fabio Fulfaro, Stefania Gori, Viviana Bazan, Antonio Russo

**Affiliations:** 1 Medical Oncology Unit, Department of Surgical, Oncological and Stomatological Sciences, University of Palermo, Via del Vespro 129, 90127 Palermo, Italy; antoniogalvano@hotmail.it (A.G.); martacastiglia@gmail.com (M.C.); sergiorizzo77@gmail.com (S.R.); giovannivaccaro@live.it (G.V.); valerio.gristina@gmail.com (V.G.); barraconadia@gmail.com (N.B.); fulfaronc@hotmail.com (F.F.); antonio.russo@usa.net (A.R.); 2 Medical Oncology Unit, IRCCS Istituto Tumori “Giovanni Paolo II” of Bari, Viale Orazio Flacco, 65, 70124 Bari, Italy; silvestrisnicola@gmail.com (N.S.); dr.oronzo.brunetti1983@gmail.com (O.B.); 3 Department of Surgical, Oncological and Stomatological Sciences, University of Palermo, Palermo, Via del Vespro 129, 90127 Palermo, Italy; marcobono29@gmail.com (M.B.); giovanni.guercio@unipa.it (G.G.); Giuseppa.graceffa@unipa.it (G.G.); 4 Oncology Department, IRCCS Sacro Cuore Don Calabria Hospital, 37024 Negrar, Verona, Italy; stefania.gori@sacrocuore.it; 5 Department of Experimental Biomedicine and Clinical Neurosciences, School of Medicine, University of Palermo, Via del Vespro 129, 90127 Palermo, Italy

**Keywords:** adjuvant, mFOLFIRINOX, pancreatic cancer, systematic review, chemotherapy, meta-analysis

## Abstract

Combination regimens have shown superiority over single agents in the adjuvant treatment of resected pancreatic cancer (PC), but there are no data supporting definition of the best regimen. This work aimed to compare the efficacy and safety of mFOLFIRINOX, gemcitabine+capecitabine, and gemcitabine+nab/paclitaxel in PC patients. A meta-analysis was performed for direct comparison between trials comparing combination regimens and gemcitabine monotherapy. Subsequently, an indirect comparison was made between trials investigating the efficacy and safety of mFOLFIRINOX, gemcitabine+capecitabine, and gemcitabine+nab/paclitaxel because of the same control arm (gemcitabine). A total of three studies met the selection criteria and were included in our indirect comparison. Indirect comparisons for efficacy outcomes showed a benefit in terms of DFS (disease-free survival)/EFS (event-free survival)/RFS (relapse-free survival) for both mFOLFIRINOX versus gemcitabine+capecitabine (HR 0.69, 95% CI 0.52–0.91) and versus gemcitabine+nab/paclitaxel (HR 0.67, 95% CI 0.50–0.90). No significant advantage was registered for OS (overall survival). Indirect comparisons for safety showed an increase in terms of G3-5 AEs (with the exception of neutropenia) for mFOLFIRINOX versus gemcitabine+capecitabine (RR 1.24, 95% CI 1.03–1.50), while no significant differences were observed versus gemcitabine+nab/paclitaxel. According to our results, mFOLFIRINOX is feasible and manageable and could represent a first option for fit PC resected patients.

## 1. Introduction

Pancreatic cancer (PC) has an annual worldwide incidence of more than 430,000 cases and an annual mortality rate of almost 460,000 cases [1]. PC has the worst 5-year survival rate of all cancers, probably because of the typically delayed diagnosis (approximately 50% of cases diagnosed in advanced stage). Even when it is diagnosed early (20% of cases) and is therefore resectable, the 5 year survival rate does not exceed 25% [2,3,4]. Consequently, surgery resection with a negative margin (R0) associated with adjuvant treatment (chemotherapy (CT) +/− radiotherapy (RT)) presents a real chance to offer a higher possibility of cure if PC can be diagnosed as early and upfront resectable disease. Due to poor long-term survival, CT is reserved for all patients undergoing pancreatic surgery, with the combination with RT suggested in cases of lymph node positivity (N+) or positive margins R1 (defined as cancer cells within 1 mm of one or more resection margins), [5]. In particular, in the last 10 years, the contribution of adjuvant chemotherapy has become significant and has been responsible for the improvement of surgery survival outcomes. Surgery guarantees about 8–10 months of survival, associated with a significant rate of local and distant recurrences [6,7]. Large randomized controlled trials (ESPAC-1 [8,9], CONKO-001 [10], ESPAC-3 [11], JASPAC-01 [12]) have investigated the efficacy and safety of 6 months gemcitabine or 5-fluorouracil (FU)/ folinic acid (FA) (or its variant used in Asian countries, named S-1) adjuvant treatment in resected PC patients, with a median overall survival (OS) of about 20 months. More recently, studies on gemcitabine- or 5-FU/FA-based combination therapies have investigated whether further intensification of chemotherapy is feasible in adjuvant settings and whether it will improve disease-free survival (DFS) and/or OS compared to the standard gemcitabine monotherapy. Three possible therapeutic options are available as new standards in the adjuvant setting: gemcitabine–capecitabine (gem-cap) [13], modified 5-FU/FA–irinitecan–oxaliplatin (mFOLFIRINOX) [14], and gemcitabine-nab/paclitaxel (gem-nab/P) [15] (the latter is under investigation by the USA Food and Drugs Administration for issues related to the choice of the primary endpoint of the study). As of the time of conception of this article, the international regulatory authorities limit the use of these combinations because of the short time elapsed since the publication of the results. Recently updated guidelines of the American Society of Clinical Oncology (ASCO) recommend 6 months of adjuvant chemotherapy following resection for PC, preferably with a combination of gemcitabine and capecitabine, and either with gemcitabine monotherapy or 5-FU/FA in cases of concerns regarding toxicity or tolerance [11]. This preferred adjuvant regimen might trouble clinicians in current clinical practice due to the lack of objective parameters from prospective randomized studies comparing the different therapeutic options. Results from retrospective comparisons between mFOLFIRINOX and gem-nab/p strategies are available only in advanced settings, and suggest no difference in efficacy [16]. In order to provide solid evidence, we analyzed and compared the efficacy and safety of adjuvant options for resected PC (gem-cap, mFOLFIRINOX, and gem-nab/P) through a systematic review of data reported in the international literature.

## 2. Results

**Figure 1:**
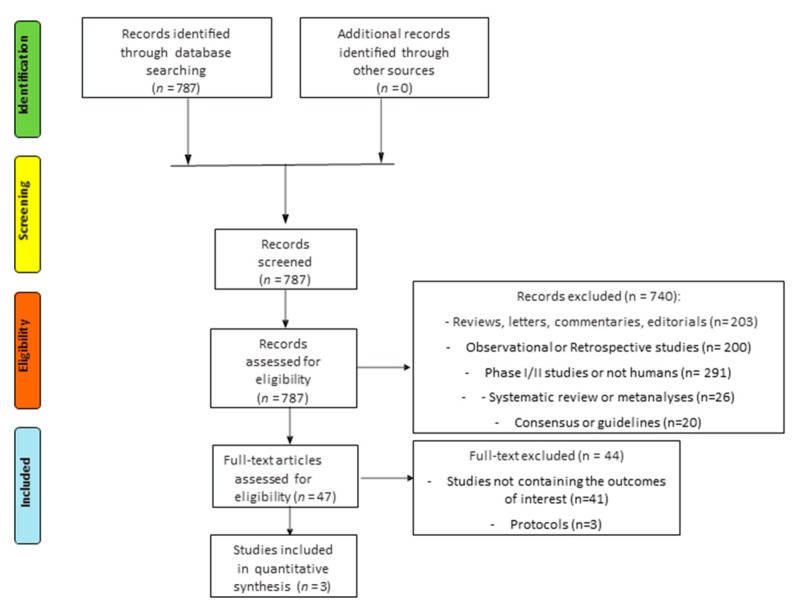
Flow diagram (CONSORT) for studies included in the meta-analysis (according to the PRISMA statement.

The search for literature identified a total of 787 records; of these, 740 records were excluded because they were systematic reviews or meta-analyses, retrospective or phase I/II studies, not human studies, consensus, or guidelines. A total of 47 trials were assessed for eligibility and 44 were excluded because no data about the principal outcomes of our indirect comparison (overall DFS/EFS/RFS, OS, discontinuation rate, grade 3–5 (G3–G5) most common AEs) were reported. Finally, a total of three studies met all the inclusion/exclusion criteria and were included in the indirect comparison (Figure 1, Table 1 and Table 2)

## 3. Combination Regimens vs. Gemcitabine Alone

**Figure 2:**
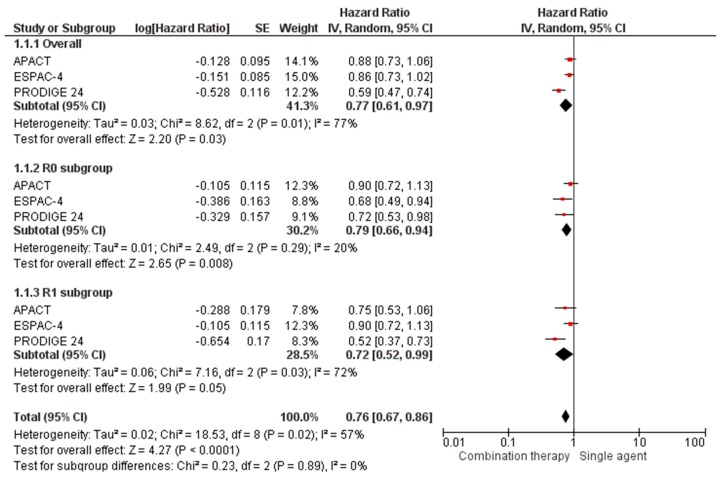
Forest plots for DFS/EFS/RFS between combination and single-agent regimens in resected PC patients.

Three RCTs enrolling 2089 patients evaluated combination regimens (gem-cap, mFOLFIRINOX, and gem-nab/P) vs. gemcitabine alone in adjuvant PC settings. Pooled results showed statistically significant differences in terms of DFS/EFS/RFS (HR 0.76, 95% CI 0.67–0.86) and OS (HR 0.79, 95% CI 0.70–0.89), favoring combination regimens. Subgroup analysis of margin status confirmed this DFS/EFS/RFS benefit in both R0 (HR 0.79, 95% CI 0.66–0.94) and R1 (HR 0.72, 95% CI 0.52–0.99) subgroups (Figure 2). As regards safety endpoints, drug combinations have been shown to significantly increase drug-related risk of G3-G5 AEs (RR 1.43, 95% CI 1.12–1.81). In particular, for the most common toxicities, an increased risk was registered for neutropenia (RR 1.25, 95% CI 1.00–1.56), fatigue (RR 2.00, 95% CI 1.01–3.97), diarrhea (RR 4.42, 95% CI 2.71–7.20), neuropathy (RR 76.44, 95% CI 10.65–548.90), and paresthesia (RR 62.28, 95% CI 3.83–1012.64). No significant differences were noted for anemia risk (RR 1.20, 95% CI 0.56–2.55). Discontinuation rate due to toxicity onset was significantly associated with combination strategy (RR 1.48, 95% CI 1.11–1.97) (see Supplementary Material, Figure S1). 

## 4. Indirect Comparisons

### 4.1. mFOLFIRINOX vs. Gemcitabine–Capecitabine

**Figure 3:**
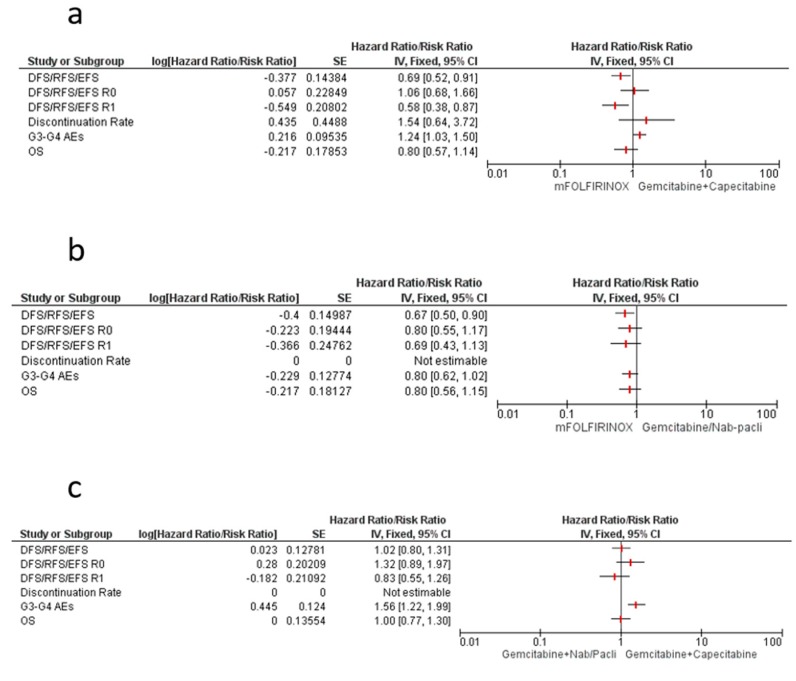
Forest plot of all indirect comparisons among combination regimens in resected PC patients: mFOLFIRINOX vs. gem-cap (**a**); mFOLFIRINOX vs. gem-nab/P (**b**); gem-nab/P vs. gem-cap (**c**). DFS/RFS/EFS: disease-free survival/relapse-free survival/event-free survival; AEs: Adverese events; OS: Overall Survival; SE: Standard error; CI: Confidence interval.

**Figure 4:**
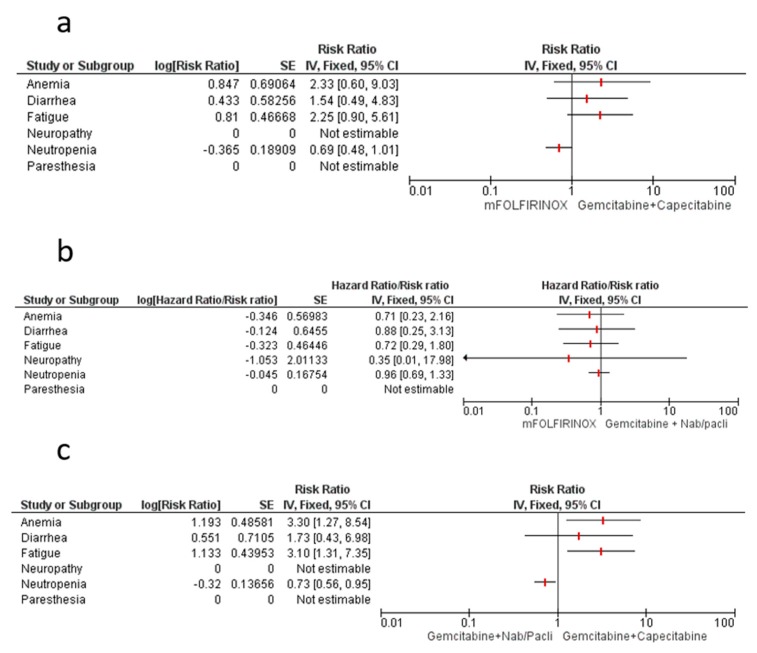
Forest plot for toxicity indirect comparisons among combination regimens in resected PC patients: mFOLFIRINOX vs. gem-cap (**a**); mFOLFIRINOX vs. gem-nab/P (**b**); gem-nab/P vs. gem-cap (**c**). DFS/RFS/EFS: disease-free survival/relapse-free survival/event-free survival; AEs: Adverese events; OS: Overall Survival; SE: Standard error; CI: Confidence interval.

We used a meta-analytic technique to make an indirect comparison between mFOLFIRINOX and gem-cap strategies in terms of clinical and safety endpoints. For clinical endpoints, mFOLFIRINOX showed a significantly better overall DFS/EFS/RFS outcome (HR 0.69, 95% CI 0.52–0.91), with a strong efficacy in the R1 patient subgroup (HR 0.58, 95% CI 0.38–0.87). No significant difference was noted in terms of OS (HR 0.80, 95% CI 0.57–1.14) (Figure 3a). In terms of the most common safety events, the mFOLFIRINOX strategy significantly increased the risk for a G3-G5 AE (RR 1.24, 95% CI 1.03–1.50), although only a non-statistically-significant trend was associated with anemia, diarrhea, and fatigue toxicities. A trend over neutropenia increase with gem-cap combination was reported and no between-treatments difference was registered in terms of discontinuation rate. Neuropathy and paresthesia differences were not estimable because of a lack of data for ESPAC-4 and APACT trials (Figure 4a).

### 4.2. mFOLFIRINOX vs. Gemcitabine–Nab/Paclitaxel

For clinical endpoints, our indirect comparison showed significantly better overall DFS/EFS/RFS for mFOLFIRINOX combination (HR 0.67, 95% CI 0.50–0.90), although no specific relationship with margin status was underlined. Only a trend towards better OS for mFOLFIRINOX was shown (HR 0.80, 95% CI 0.56–1.15) (Figure 3b). Regarding the most common safety events, mFOLFIRINOX strategy showed a trend towards reduced risk for a G3–G5 AE (RR 0.80, 95% CI 0.62–1.02), and non-statisticallysignificant differences were associated with anemia, diarrhea, fatigue, neuropathy, and neutropenia. Paresthesia and discontinuation rate were not evaluated because of a lack of data for the APACT trial (Figure 4b).

### 4.3. Gemcitabine–Nab/Paclitaxel vs. Gemcitabine–Capecitabine

As regards clinical endpoints, our indirect comparison showed no between-treatment difference in terms of overall DFS/EFS/RFS (HR 1.02, 95% CI 0.80–1.31), although an improved trend for R0 was shown for the gem-cap combination, while the gem-nab/p combination showed a better efficacy trend in the R1 subgroup. No OS difference was registered (Figure 3c). Regarding the most common safety events, the gem-nab/p strategy was associated with a significantly increased risk for a G3–G5 AE (RR 1.56, 95% CI 1.22–1.99), particularly anemia (RR 3.30, 95% CI 1.27–8.54) and fatigue (RR 3.10, 95% CI 1.31–7.35). Only a trend was registered for fatigue. 

The gem-cap combination was associated with a reduced risk for neutropenia (RR 0.73, 95% CI 0.56–0.95). 

Paresthesia, neuropathy, and discontinuation rate were not evaluated because of a lack of data for the APACT and ESPAC-4 trials (Figure 4c).

## 5. Risk of Bias Assessment (JADAD Scale)

**Figure 5:**
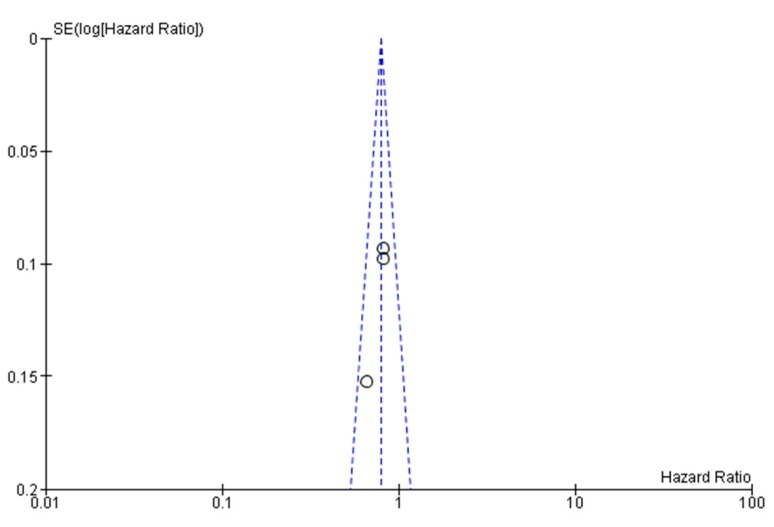
Publication bias assessment (funnel plot).

A publication bias test is necessary in a meta-analysis including at least three studies. In our analysis, Egger’s test was conducted on the three evaluated trials comparing combination regimens vs gemcitabine alone, showing no statistical significance (*p* = 0.27) (funnel plot, Figure 5). The overall quality assessment was conducted according to the CONSORT checklist statement. We reported an average good quality of all trials. Some problems related to the “blinding of participants and personnel (performance bias)” domain were noted because all evaluated publications were open-label studies. Risk were unclear for the APACT trial, because this study had not been published in its full text at the time of publication of our manuscript (see Supplementary Material, Figure S2).

## 6. Discussion

Despite all the available therapeutic options, PC remains the neoplasm with the worst prognosis, even if diagnosed at an early stage. This unfavorable trend in developed countries is supported by late diagnosis, early spreading at a distance, and poor sensitivity to chemo-radiotherapy treatments [10,17,18]. Furthermore, only about half of patients undergo post-operative adjuvant treatment due to post-operative clinical conditions and modest performance status [19,20,21]. Therefore, the main aim of an adjuvant regimen is a favorable ratio between benefits (decrease in relapse rate, OS prolongation) and costs (toxicity, financial health). Although gemcitabine has been considered the best option in this setting in recent years, some new chemotherapeutic combinations have been developed based on great results obtained in palliative settings. Despite this, no data from comparative studies between the different options or international guidelines can suggest an effective criterion for selecting the correct adjuvant strategy for an individual patient. The aim of this indirect comparison was to evaluate the impact of combination regimes in radically operated PC, and also to assess whether a preferable regimen exists. First, from our results emerged the finding that the combination strategy has shown a clear benefit in terms of DFS/EFS/RFS with no significant impact on margin status. The significant impact of these results translates into a 21% reduction in global mortality and in a 43% increase in the risk of developing severe toxicities. 

mFOLFIRINOX consists of a kind of FOLFIRINOX (fluorouracil bolus suppression, irinotecan dose reduction) investigated to decrease hematological and non-hematological effects (especially nausea and vomiting). In the PRODIGE-24 study, patients randomly underwent a modified FOLFIRINOX regimen or a gemcitabine regimen for 24 weeks. The modified FOLFIRINOX regimen consisted of oxaliplatin 85 mg/m^2^, leucovorin 400 mg/m^2^, irinotecan 150 mg/m^2^, and continuous fluorouracil 2400 mg/m^2^, without bolus fluorouracil. However, mFOLFIRINOX remains a protocol with many side effects, so it could be administered especially to fit patients [22,23], since in the adjuvant setting there are no serological alterations (such as an increase in bilirubin) which may affect the use of irinotecan [24]. Certainly, the choice of adjuvant treatment can also affect the choice of first-line treatment, since the appearance of oxaliplatin neuropathy makes it difficult to subsequently use a combination with nab-paclitaxel. Despite these limitations, our results suggest for the first time that mFOLFIRINOX may be an appropriate therapeutic standard in patients with good PS (0–1 ECOG) after surgery, despite some potential limitations in interpreting the surprising OS results of the gemcitabine control arm in the PRODIGE-24 study—probably due to the use of mFOLFIRINOX and other active regimens after disease recurrence [25], since similar DFS excludes the possibility of selection bias. Clinical parameters could be used to select patients for mFOLFIRINOX approach. Recently, Schlick et al [26] evaluated 123 metastatic PC (mPC) patients who underwent FOLFIRINOX treatment, showing by multivariate analysis that BMI > 25 and CEA > 4 at the time of onset of advanced disease could be interpreted as independent negative prognostic factors for OS and for toxicity, also suggesting a role for clinical parameters in patient selection. Other parameters such as the neutrophil-to-lymphocyte ratio (NLR), a marker of systemic inflammation, could be considered for selection, but further clinical studies are needed to support this [27], especially in an adjuvant setting.

In the ESPAC-4 study, the gem-cap cohort received adjuvant gemcitabine (at the standard dose of 1000 mg/m^2^) in association with oral capecitabine at the dose of 830 mg/m^2^ twice a day on days 1-21/28) for a total of six cycles. The gem-cap combination aimed to harness the synergistic antitumor activity of gemcitabine and capecitabine, with its peculiar pharmacokinetic activities in intestine, able to produce higher levels of 5-fluorouracil in the tumor microenvironment after a three step enzymatic conversion while limiting systemic side effects. The use of capecitabine, although not supported by significant benefits in clinical studies, is considered reasonable because of improved patient quality of life outcomes, as well as the absence of valid therapeutic alternatives. Furthermore, these favorable properties of capecitabine suggest that its efficacy and safety should be tested within the mFOLFIRINOX regimen in place of the 5-FU/FA infusion, especially since the SOXIRI regimen (S-1, oxaliplatin, and irinotecan) is showing promising results in metastatic PC [28]. Although our analysis showed significant superiority of the mFOLFIRINOX regimen compared to the gem-cap combination in terms of DFS/EFS/RFS, especially in R1 patients (60%), the superior toxicity profile of mFOLFIRINOX (with the exception of neutropenia) may not recommend its use in patients with less favorable performance status or with comorbidity. In addition, the gem-cap cohort contained more unfavorable prognosis patients (locally advanced, large or not completely removed tumors), and was probably more representative of the real-world experience. 

The addition of nab-paclitaxel to gemcitabine could constitute a new therapeutic option for radically resected patient PC. In the APACT trial, patients were randomized to receive six cycles of either nab-paclitaxel 125 mg/m^2^ plus gemcitabine 1000 mg/m^2^, or gemcitabine alone at 1000 mg/m^2^ on Days 1, 8, and 15 of a 4 week cycle. The choice of primary endpoint of the phase III APACT study (independently assessed DFS based on radiographic review) may have affected the overall result of the study and therefore the registration of the protocol. However, our analysis suggested that the gem-nab/P combination is inferior in terms of DFS/EFS/RFS if compared with the mFOLFIRINOX regimen, but superior to the gem-cap regimen in patients with R1 surgery. Gem-nab/P also demonstrates a toxicity profile similar to mFOLFIRINOX (with the exception of neuropathy), although it is more toxic compared to the combination with capecitabine (with the exception of neutropenia). In the next few years, much more information will probably come out regarding the prognosis and the efficacy of these new regimens as a consequence of the advent of liquid biopsy techniques. In fact, liquid biopsy allows traditional limitations of tissue biopsy linked to spatial and temporal heterogeneity to be overcome, with the advantage of being less invasive and easily repeatable. Extensive evidence suggests that high levels of circulating tumor DNA (ctDNA) may predict disease progression in mPC [29,30,31]. To investigate ctDNA in an adjuvant setting could help to select patients with minimal residual disease at risk of post-surgical recurrence, probably even better than the use of circulating tumor cells (CTC) or CA19.9, although nowadays definitive data are not disposable [32,33,34]. It is desirable that in the near future, these biological factors could be used to select patients on whom to use adjuvant chemotherapy, preventing unnecessary toxicity and saving costs for patients and health systems.

Certainly, despite the above-mentioned improvements, PC management remains difficult, linked to the patients’ clinical conditions after surgery. In fact, up to 50% of patients develop major post-operative complications that affect the adjuvant chemotherapy completion rate (around 54–79% in highly selected patients from randomized trials) [13,14,35,36,37]. Furthermore, early relapse affects about one third of patients, making the administration of preventive chemotherapy unnecessary [38,39]. A recent retrospective analysis of about 2500 radically operated PC showed that the small proportion of patients (about 10%) who completed the adjuvant chemotherapy protocol reported better survival outcomes (22 months) than those who partially completed the protocol (17 months) or who did not have chemotherapy (14 months). Our indirect comparison results underlined that mFOLFIRINOX has a higher discontinuation rate (trend) compared to the gem-cap combination, suggesting great benefits of mFOLFIRINOX for those selected patients able to complete the chemotherapy protocol [40], although data concerning the combination with nab/P have not yet been reported. Our analysis carried some limitations linked to the modest number of trials available with which to compare the three chemotherapeutic regimens, the enrolled populations (the gem-cap combination group had high numbers of PS1, R1, and node-positive patients; mFOLFIRINOX high CA.19 level) and the different primary endpoints (DFS/RFS/EFS for mFOLFIRINOX and gem-nab/P and OS for gem-cap). In our opinion, the different rate of R1 patients and the good performance of the PRODIGE-24 gemcitabine control group could explain overall survival trend identified in our analysis, because of high rate of other active regimens (e.g., FOLFIRINOX) used after disease progression.

To our knowledge, our indirect comparison is the first valid attempt to provide robust evidence in a PC adjuvant setting supporting an mFOLFIRINOX regimen as a preferable therapeutic approach in fit patients. In fact, despite the already known severe toxicity profile of mFOLFIRINOX, a recent systematic review of the literature with meta-analysis [41] established that mFOLFIRINOX produces similar survival benefits with reduced adverse event rates. Similar results have been obtained from "real-world" experiences [42]. Despite this, it is desirable to collect data in the future about the efficacy and safety of these new regimens in an unselected population of real-world PC resected patients in order to highlight any significant differences between this context and the registrational trials. The combination gemcitabine-based regimens with the addition of capecitabine or nab/paclitaxel should be considered two valid therapeutic alternatives in patients judged unfit for mFOLFIRINOX (nab/paclitaxel if approved by international regulatory agencies). In patients with an unfavorable performance status not eligible for combination therapy, treatment with gemcitabine or 5-fluorouracil monotherapy for 6 months can be considered a reasonable option. Several questions regarding the possible use of mFOLFIRINOX in combination with radiotherapy or as a neoadjuvant treatment should be investigated. 

## 7. Materials and Methods

### 7.1. Search for Trials

We searched for results of randomized controlled trials (RCTs) comparing combination regimens with gemcitabine alone, historically considered the standard adjuvant regimen in patients with histologically proven diagnoses of PC. Data available up to November 2019 on Medline (PubMed (https://www.ncbi.nlm.nih.gov/pubmed/, accessed on 15 December 2019)), EMBASE databases, Cochrane-Library, and TCGA database were collected, without language restrictions; relevant abstracts published on the ASCO and the European Society of Medical Oncology (ESMO) databases, as well as unpublished data or results from ongoing studies available on the National Institute of Health (NIH) website (www.clinicaltrials.gov) were also considered as a source of grey literature. We used the following free term strategy: “pancreatic”, “adjuvant”, “resected”, “gemcitabine”, “chemotherapy”, “drug therapy”, and “pancreatic neoplasm”. We produced the following search string: ((“Pancreatic Neoplasms”[Mesh] OR “pancreatic”[tiab]) AND (“adjuvant”[tiab] OR “resected”[tiab]) AND (“gemcitabine”[Supplementary Concept] OR “gemcitabine”[tiab] OR “gem”[tiab]) AND (“drug therapy”[MeSH Terms] OR “chemotherapy”[tiab])). 

### 7.2. Selection Criteria

The outcomes were DFS (disease-free survival), EFS (event-free survival), RFS (relapse-free survival), OS (overall survival), and discontinuation rate. R0 surgical margin was defined as absence of tumor cells within 1 mm of all resection margins; R1 surgical margin was defined as presence of tumor cells within 1 mm of one or more resection margins (microscopic positive margin). Safety data (AEs—adverse events) were included in our analysis according to common terminology criteria for adverse event (CTCAE) grading. We excluded non-phase III randomized or cross-sectional, case–control, cohort, or retrospective studies and studies assessing the role of chemo-radiation or targeted therapies in an adjuvant setting. We also excluded trials in which outcome data were unavailable, ongoing studies, and studies with small sample sizes (fewer than 10 patients per arm). 

### 7.3. Data Extraction and Risk of Bias Assessment

To minimize the risk of bias, we excluded observational trials. In the case of articles with follow-ups published over time, we decided to include the most updated and methodically valid one. Two review authors (A.G. and N.S.) independently screened articles for inclusion and were responsible for data extraction and assessment. Details about study structure, participants, rules, efficacy, and safety were recorded. Incongruences and disagreements were solved by discussing with another author (A.R.). We made a quality analysis of selected trials following the criteria reported in the Cochrane Handbook for Systematic Reviews of Interventions [43] including allocation concealment, blinding of participants, personnel and outcome assessors, incomplete outcome data, sequence generation, elective outcome reporting, and other sources of bias. For each study, we defined “Yes” as being at low risk of bias and “No” as being at high risk of bias. We also defined “unclear” if there were insufficient data to make a precise judgment. The risk of selective outcome reporting bias was also evaluated by two independent reviewers (A.G. and N.S.) and disagreements were solved by consensus.

### 7.4. Assessment of Heterogeneity and Statistical Analysis

The analyzed outcomes for both direct and indirect comparisons were overall DFS/EFS/RFS, OS, discontinuation rate, and grade 3–5 (G3–G5) most common AEs. In addition, DFS/EFS/RFS was stratified, when possible, according to surgical margin (R0 or R1) status. We used hazard ratios (HRs) to assess the associations for DFS/EFS/RFS and OS, with a 95% confidence interval (CI). For all other outcomes (AEs, discontinuation rate), we calculated the total number of events over the total patients randomized in each group, thus using risk ratios (RRs) as the measure of association. In the first phase of the study, using meta-analysis techniques, we performed a direct comparison including all the RCTs evaluating the efficacy of combination therapy (gem-cap, mFOLFIRINOX, and gem-nab/P) versus standard gemcitabine alone, by calculating the logarithm of the HR (logHRs) or RR (logRRs) and their relative standard error (SE) for all the RCTs included in this phase. Afterwards, we calculated the pooled data of every comparison.

We took final estimates from direct comparisons in order to obtain the pooled estimates of HR and RR needed for the indirect comparison [44]. The method used in the indirect comparison was the one described by Bucher and Glenny, extended to calculate the HR [45,46]. We chose this method for its ability to maintain the randomization advantage of each trial, providing an estimate of the comparison between treatments. Assuming that mFOLFIRINOX_ST_ is the estimate of the direct comparison between mFOLFIRINOX and gemcitabine, gem-cap_ST_ is the estimate of the direct comparison between gem-cap and gemcitabine, and the estimate of the indirect comparison between mFOLFIRINOX and gem-cap can be calculated as follows: mFOLFIRINOX/gem-cap_indirect (logHR or logRR) = mFOLFIRINOX_ST_ (logHR or logRR) − gem-cap_ST_ (logHR or logRR).

The variance can be obtained with the following computation: Var (log mFOLFIRINOX/gem-cap_indirect) = Var(log mFOLFIRINOX_ST_) + Var(log gem-cap_ST_) [47]. The mFOLFIRINOX versus gem-cap heterogeneity between studies was evaluated using an I-squared test. If the I-squared value was higher than 50%, with a high risk of heterogeneity, we performed the meta-analysis using the random effect-based model by Der Simonian and Laird; for I-squared values lower than 50%, we used the fixed-effect-based Mantel–Haenszel mode [46,48]. In our indirect meta-analysis, for example, outcomes with HR < 1 suggested a better efficacy of mFOLFIRINOX, whereas outcomes with RR < 1 suggested a better toxicity profile of the gem-cap regimen. The same statistical considerations were used to make the mFOLFIRINOX versus Gem-nab/P and Gem-nab/P versus gem-cap comparisons. As regards the between-studies bias, we performed a publication bias test using Egger’s test, providing the associated funnel plot. The manuscript was done and reported according to the PRISMA guidelines for reporting on systematic reviews [49]. The meta-analysis was performed using Cochrane RevMan ver. 5.3 software [50], and *p*-values were considered statistically significant if *p* < 0.05. 

## 8. Conclusions

Our results confirmed a significant advantage in terms of DFS/EFS/RFS in the R1 cohort of mFOLFIRINOX compared to the two gem-based combinations, and therefore strongly support the pre-operative use of mFOLFIRINOX to increase the chance of R0 resection and reduce the incidence of micro-metastases. The study of predictive biomarkers predictive could likely guide clinicians in the near future to select the right patients for intensive chemotherapy treatment with mFOLFIRINOX [51,52].

## Figures and Tables

**Table 1:** Characteristics of the Trials Included in the Indirect Comparison.

Study (Reference)	Drug	*n*	DFS/RFS/EFS HR (95% CI)	OS HR (95% CI)	G3–G5 AEs *n*. (%)	Discontinuation Rate *n*. (%)	R1 DFS/RFS/EFS HR (95% CI)	R0 DFS/RFS/EFS HR (95% CI)
Neoptolemos J.P.et al (ESPAC-4) [13]	Gem + Cape	364	0.86 (0.73–1.02)	0.82 (0.68–0.98)	226/359 (63)	11/364	0.90 (0.72–1.13)	0.68 (0.49–0.93)
	Gem	366			199/366 (54)	11/366		
Tampero M.A. et al (APACT) [15]	Gem + Nab-P	432	0.88 (0.73–1.06)	0.82 (0.68–1.00)	176/429	NA	0.75 (0.53–1.07)	0.90 (0.72–1.13)
	Gem	434			96/423			
Conroy T. et al (PRODIGE 24) [14]	Folfirinox	247	0.59 (0.47–0.74)	0.66 (0.49–0.89)	180 (75.5)	80/247	0.52 (0.37–0.72)	0.72 (0.53–0.98)
	Gem	246			128 (51.1)	51/246		

**Table 2:** Side-effects rates of the Trials included in the Indirect Comparison.

Study (Reference)	Drug	Neutropenia *n*. (%)	Anemia *n*. (%)	Fatigue *n*. (%)	Diarrhea *n*. (%)	Neuropathy *n*. (%)	Paresthesia *n*. (%)
Neoptolemos J.P. et al. (ESPAC-4) [13]	Gem + Cape Gem	137 (38) 89 (24)	8 (2) 14 (4)	20 (6) 19 (5)	19 (5) 6 (2)	NA	NA
Tampero M.A. et al. (APACT) [15]	Gem + Nab-P Gem	212 (49) 184 (43)	63 (14) 33 (8)	43 (10) 13 (3)	22 (5) 4 (1)	64 (15) 0 (0)	NA
Conroy T. et al. (PRODIGE 24) [14]	Folfirinox Gem	67/238 (28.4) 63/243 (26)	8 (3.4) 6 (2.5)	26 (11) 11 (4.6)	44 (18.6) 9 (3.7)	22 (9.3) 0 (0)	30 (12.7) 0 (0)

ORR: overall response rate; PFS: progression-free survival; OS: overall survival; PD-L1: Programmed death-ligand 1; *n*.: number; HR: hazard ratio; CI: confidence interval; NA: not available.

## Supplementary Materials

The following are available online at https://www.mdpi.com/2072-6694/12/3/534/s1, Figure S1: Forest plots for toxicity between combination and single-agent regimens in resected PC patients. Figure S2: Risk of bias graph: review author’s judgements about each risk of bias item presented as percentages across all included studies (a); Risk of bias summary: review author’s judgements about each risk of bias item for each included studies.
